# Inferring Species Compositions of Complex Fungal Communities from Long- and Short-Read Sequence Data

**DOI:** 10.1128/mbio.02444-21

**Published:** 2022-04-11

**Authors:** Yiheng Hu, Laszlo Irinyi, Minh Thuy Vi Hoang, Tavish Eenjes, Abigail Graetz, Eric A. Stone, Wieland Meyer, Benjamin Schwessinger, John P. Rathjen

**Affiliations:** a Research School of Biology, Australian National University, Canberra, ACT, Australia; b Molecular Mycology Research Laboratory, Centre for Infectious Diseases and Microbiology, Faculty of Medicine and Health, Sydney Medical School, Westmead Clinical School, The University of Sydney, Sydney, NSW, Australia; c Sydney Institute for Infectious Diseases, The University of Sydney, Sydney, NSW, Australia; d Westmead Institute for Medical Research, Westmead, NSW Australia; e ANU-CSIRO Centre for Genomics, Metabolomics and Bioinformatics, Canberra, ACT, Australia; f Westmead Hospital (Research and Education Network), Westmead, NSW, Australia; g Curtin Medical School, Curtin University, Perth, Bentley, WA, Australia; Cornell University

**Keywords:** bioinformatics, fungi, metagenomics, pathogens

## Abstract

The kingdom Fungi is highly diverse in morphology and ecosystem function. Yet fungi are challenging to characterize as they can be difficult to culture and morphologically indistinct. Overall, their description and analysis lag far behind other microbes such as bacteria. Classification of species via high-throughput sequencing is increasingly becoming the norm for pathogen detection, microbiome studies, and environmental monitoring. With the rapid development of sequencing technologies, however, standardized procedures for taxonomic assignment of long sequence reads have not yet been well established. Focusing on nanopore sequencing technology, we compared classification and community composition analysis pipelines using shotgun and amplicon sequencing data generated from mock communities comprising 43 fungal species. We show that regardless of the sequencing methodology used, the highest accuracy of species identification was achieved by sequence alignment against a fungal-specific database. During the assessment of classification algorithms, we found that applying cutoffs to the query coverage of each read or contig significantly improved the classification accuracy and community composition analysis without major data loss. We also generated draft genome assemblies for three fungal species from nanopore data which were absent from genome databases. Our study improves sequence-based classification and estimation of relative sequence abundance using real fungal community data and provides a practical guide for the design of metagenomics analyses focusing on fungi.

## INTRODUCTION

Fungi are ubiquitous yet their presence and impact are often overlooked. It has been estimated that 2.2–3.8 million fungal species inhabit planet earth (1) but only about 4% of these are catalogued (2). Fungi play diverse and fundamental roles throughout evolution. For example, saprotrophs break down dead organic matter to release nutrients, whereas mycorrhizae facilitate nutrient uptake by plants in soil. Fungi constitute a major disease load to humans, causing millions of deaths per year, and wreak devastating crop losses via a constant toll of disease and are an existential threat to many plant species (3, 4). On the other hand, fungi are or are used to manufacture delicious foods and beverages and have saved countless lives via antibiotic production (5, 6). Therefore, a call was made recently to expand fungal research and improve our awareness of this special kingdom (7).

To progress our understanding of fungal biology we need to improve our ability to correctly identify species from complex mycological samples. Historically, taxonomic assignment was based on morphological and reproductive traits, but this has been largely surpassed by DNA-based classification which revolutionized mycology, not only refining the conventional taxonomic tree (8, 9) but also standardizing the identification of species. In the absence of whole-genome data, DNA-based classification primarily exploits the internal transcribed spacer (ITS) within the rRNA genes as a highly polymorphic marker to distinguish species, being designated the primary fungal barcode (10). It is easily amplified and sequenced due to highly conserved flanking sequences and contains a high degree of variation between even closely related species. Although a mature pipeline comprising ITS amplification, IIlumina sequencing and data analysis has been established (11), several studies reported biases from the sequencing technology used and from unevenly amplified fungal marker regions (12–14). Recently, novel strategies exploiting long-range amplification and long-read sequencing have been developed to improve these classifications (15, 16). In addition, whole-genome shotgun sequencing and rapidly expanding genome databases allow mapping of newly generated DNA sequences directly to the database. This so-called shotgun metagenomics strategy has now been applied widely to dissect the structure and composition of complex microbial communities. Shotgun metagenomics also allows exploitation of genetic variation throughout the genome and abandonment of the marker gene amplification step, which increases classification accuracy and reduces the biases in estimation of relative abundance (17).

Although advanced sequencing methods allow novel strategies for fungal identification particularly from mixed samples, new demands are placed on data analysis pipelines to improve the accuracy of sequence classification. Various algorithms have been developed to classify DNA sequences at distinct taxonomic ranks based on sequence databases containing taxonomic information (18–22). For example, alignment algorithms such as Basic Local Alignment Search Tool (BLAST) (19) detect matches of each sequence to subjects of the target database along with the taxonomic information assigned to each entry. Alternatively, sequence features represented by short unique subsequences named k-mers can be derived from sequence data and mapped to databases to identify taxa with the highest number of cross-mapping k-mers (20). Several studies have critically assessed algorithms for species classification on simulated data sets or bacterial community data sets (23–25), but benchmarking of long read sequencing strategies for taxonomic classification of fungal communities using real data are extremely rare. As such, the bioinformatic tools to process fungal sequencing data, particularly long reads, are not as well developed as those for bacteria. In addition to bioinformatic tools, the choice of database also influences classification dramatically, but only a few studies have researched their impact (26–28). Compared to bacterial genomes, fungi contain complex genetic features such as multiple chromosomes, expanded repeat regions and larger genome sizes, all of which introduce inaccuracies during sequence classification. Therefore, comprehensive benchmarking of both classification algorithms and databases is needed to optimize identification pipelines for the kingdom fungi.

Here, we assessed different combinations of algorithms and databases during processing of both short- and long-read sequencing data for the identification of taxa from mock fungal communities. We constructed a low diversity community consisting of ascomycetous and basidiomycetous fungal species, which were designed to mimic fungal identification scenarios from clinical or other host-associated environmental samples. We identified key factors that influence the accuracy of classifications, both for mock community data sets and public data sets. Optimization of these methods lead to more accurate community compositions. Our results provide guidelines for the design of sequence-based community analysis for fungal species.

## RESULTS

### Construction of mock fungal community data sets.

We constructed two mock communities from the same set of 43 fungal species (Table 1), consisting of yeasts and filamentous human-associated pathogens. We aimed to mimic a real-world situation with a reasonable level of species diversity in order to adjust different technical search parameters while aiming to recover all species. One community comprised pooled DNA individually extracted from each species and the second was composed of DNA extracted from equal quantities of fungal biomass (pooled biomass) of each species mixed together prior to the extraction. We generated four sequence data sets for each community using Illumina and nanopore technologies, sequencing both shotgun metagenomes and targeted amplicons, respectively. The data derived from each strategy are summarized in Table 2.

**TABLE 1:** Metadata of the mock fungal community

Species name	Strain used in the mock community	Corresponding reference genome strain	Assembly level	NCBI accession	File name
Aspergillus flavus	WM 03.225	NRRL3357	Scaffold	GCF_000006275.2	GCF_000006275.2_JCVI-afl1-v2.0_genomic.fna.gz
Aspergillus fumigatus	WM 06.98	Af293	Chromosome	GCF_000002655.1	GCF_000002655.1_ASM265v1_genomic.fna.gz
*Blastobotrys proliferans*	WM 07.12 (CBS 522.75 Type strain)	NRRL Y-17577	Scaffold	GCA_003707485.2	GCA_003707485.2_ASM370748v2_genomic.fna.gz
Candida albicans	WM 229 (CBS 562 Type strain)	SC5314	Chromosome	GCA_000182965.3	GCA_000182965.3_ASM18296v3_genomic.fna.gz
Candida dubliniensis	WM 02.73	CD36	Chromosome	GCA_000026945.1	GCA_000026945.1_ASM2694v1_genomic.fna.gz
Candida glabrata	WM 03.500	CBS 138	Chromosome	GCF_000002545.3	GCF_000002545.3_ASM254v2_genomic.fna.gz
*Candida haemulonis*	WM 890	B11899	Contig	GCF_002926055.2	GCF_002926055.2_CanHae_1.0_genomic.fna.gz
Candida parapsilosis	WM 02.200	CDC317	Scaffold	GCA_000182765.2	GCA_000182765.2_ASM18276v2_genomic.fna.gz
Candida tropicalis	WM 01.203	MYA-3404	Scaffold	GCF_000006335.3	GCF_000006335.3_ASM633v3_genomic.fna.gz
Clavispora lusitaniae	WM 18 (CBS 4413 Type strain)	ATCC 42720	Scaffold	GCF_000003835.1	GCF_000003835.1_ASM383v1_genomic.fna.gz
*Naganishia albida (former* Cryptococcus albidus*)*	WM 773 (CBS 142 Type strain)	JCM 2334	Scaffold	GCA_001599735.1	GCA_001599735.1_JCM_2334_assembly_v001_genomic.fna.gz
Cryptococcus gattii *VGI*	WM 179 (VGI Standard strain)	WM 276	Chromosome	GCF_000185945.1	GCA_000185945.1_ASM18594v1_genomic.fna.gz
Cryptococcus gattii *VGII*	WM 178 (VGII Standard strain)	R265	Scaffold	GCA_000786445.1	GCA_000786445.1_R265.1_genomic.fna.gz
Cryptococcus gattii *VGIII*^*a*^	WM 175 (VGIII Standard strain)				
Cryptococcus gattii *VGIV*^*a*^	WM 779 (VGIV Standard strain)				
*Filobasidium magnum (former* Cryptococcus *magnus)*^*b*^	WM 13.104				
Cryptococcus neoformans *VNI*	WM 148 (VNI Standard strain)	JEC21	Chromosome	GCA_000091045.1	GCA_000091045.1_ASM9104v1_genomic.fna.gz
Cryptococcus neoformans *VNII*	WM 626 (VNII Standard strain)	B-3501A	Chromosome	GCF_000149385.1	GCF_000149385.1_ASM14938v1_genomic.fna.gz
Cryptococcus neoformans *VNIV*	WM 629 (VNIV Standard strain)	H99	Chromosome	GCF_000149245.1	GCF_000149245.1_CNA3_genomic.fna.gz
*Cyberlindnera jadinii*	WM 45 (CBS 1600 Type strain)	NBRC0988	Chromosome	GCA_000328385.1	GCA_000328385.1_Cuti_1.0_genomic.fna.gz
Debaryomyces hansenii	WM 309 (CBS 767 Type strain)	CBS767	Chromosome	GCF_000006445.2	GCF_000006445.2_ASM644v2_genomic.fna.gz
*Diutina catenulata (former* Candida catenulata*)*^*b*^	WM 03.477	WY3-10-4	Contig	GCA_003285555.1	GCA_003285555.1_ASM328555v1_genomic.fna.gz
*Diutina mesorugosa (former Candida mesorugosa)* ^*b*^	WM 03.468				
*Diutina rugosa (former Candida rugosa)* ^*b*^	WM 03.463				
Geotrichum candidum	WM 05.217	CLIB 918	Scaffold	GCA_001402995.1	GCA_001402995.1_New2.3_08062011_genomic.fna.gz
*Geotrichum fermentans*	WM 17.18	CICC 1368	Scaffold	GCA_002233575.1	GCA_002233575.1_ASM223357v1_genomic.fna.gz
Kluyveromyces marxianus	WM 13 (CBS 834)	DMKU3-1042	Complete Genome	GCF_001417885.1	GCF_001417885.1_Kmar_1.0_genomic.fna.gz
*Kodamaea ohmeri*	WM 10.200	148	Contig	GCA_004919595.1	GCA_004919595.1_Kodohm_148_genomic.fna.gz
*Lomentospora prolificans*	WM 13.369	JHH-5317	Contig	GCA_002276285.1	GCA_002276285.1_Lprolificans_pilon_genomic.fna.gz
*Meyerozyma caribbica*	WM 03.389	MG20W	Contig	GCA_000755205.1	GCA_000755205.1_ASM75520v1_genomic.fna.gz
Meyerozyma guilliermondii	WM 02.131	ATCC 6260	Scaffold	GCF_000149425.1	GCF_000149425.1_ASM14942v1_genomic.fna.gz
*Pichia kudriavzevii*	WM 02.78	CBS573	Complete Genome	GCA_003054445.1	GCA_003054445.1_ASM305444v1_genomic.fna.gz
*Pichia membranifaciens*	WM 32 (CBS 638 Type strain)	NRRL Y-2026	Scaffold	GCF_001661235.1	GCF_001661235.1_Picme2_genomic.fna.gz
*Pichia norvegensis*	WM 885	NRRL Y-7687	Scaffold	GCA_003705465.1	GCA_003705465.1_ASM370546v1_genomic.fna.gz
Purpureocillium lilacinum ^*c*^		PLFJ-1		GCA_004026455.1	GCA_004026455.1_ASM402645v1_genomic.fna
Rhodotorula mucilaginosa	WM 09.204	RIT389	Contig	GCA_002250355.1	GCA_002250355.1_RIT389_v1_genomic.fna.gz
*Scedosporium aurantiacum*	WM 06.385	WM 09.24	Scaffold	GCA_000812075.1	GCA_000812075.1_ASM81207v1_genomic.fna.gz
Scedosporium boydii *(former* Pseudallescheria boydii*)*	WM 09.122 (CBS 101.22 Type strain)	IHEM 23826	Contig	GCA_002221725.1	GCA_002221725.1_ScBoyd1.0_genomic.fna.gz
Trichophyton rubrum	WM 04.474	CBS 118892	Scaffold	GCF_000151425.1	GCF_000151425.1_ASM15142v1_genomic.fna.gz
*Trichosporon asahii*	WM 03.423	CBS 2479	Scaffold	GCF_000293215.1	GCF_000293215.1_Trichosporon_asahii_1_genomic.fna.gz
*Cutaneotrichosporon dermatis (former Trichosporon dermatis)*	WM 601	JCM 11170	Scaffold	GCA_003116895.1	GCA_003116895.1_JCM_11170_assembly_v001_genomic.fna.gz
*Wickerhamomyces anomalus*	WM 03.507	NRRL Y-366-8	Scaffold	GCF_001661255.1	GCF_001661255.1_Wican1_genomic.fna.gz
Yarrowia lipolytica	WM 17 (CBS 6124 Type strain)	CLIB122	Chromosome	GCF_000002525.2	GCF_000002525.2_ASM252v1_genomic.fna.gz
*Zygoascus hellenicus* ^*d*^	WM 02.460 (CBS 5839 Type strain)	Y-7136	Scaffold		Zyghe1_2_AssemblyScaffolds_Repeatmasked.fasta

a No publicly available reference genome, but high quality reference genomes of relative strains.

b Locally sequenced and assembled.

c Potential contamination species.

d Downloaded from JGI, no NCBI accession.

**TABLE 2:** Characteristics of each sequence data set

Sample	Sequencing tech	Sequencing strategy	Number base pairs	Number reads	Number assembled contigs	Number mapped base pairs (Gb)
Pooled DNA	Illumina	Shotgun	3.91 Gb	14,525,058	338,823	3.69
		Amplicon	66.9/95.8/106.4 Mb^*a*^	39,374/9,614/10,236^*b*^	NA	N/A
	Nanopore	Shotgun	1.96 Gb	1,273,484	NA	N/A
		Amplicon	71.5/72.5/86.5 Mb	26,212/ 26,680/ 31,826^*b*^	NA	N/A
Pooled biomass	Illumina	Shotgun	3.67 Gb	13,623,120	345,009	3.44
		Amplicon	55.7/38.1/71.9 Mb^*a*^	23,613/13,828/27,093^*b*^	NA	N/A
	Nanopore	Shotgun	3.78 Gb	1,043,343	NA	N/A
		Amplicon	54.5/49.4/42.0 Mb	20,163/ 18,273/ 15,502^*b*^	NA	N/A

a The total number of basepairs of each technical replicate was calculated before import into QIIME2 pipeline.

b Number of nanopore reads or paired-end Illumina reads for technical replicate 1/replicate 2/replicate 3 after quality control.

### Alignment algorithm against a fungal-specific database resulted in the most accurate fungal classification.

**FIG 1:**
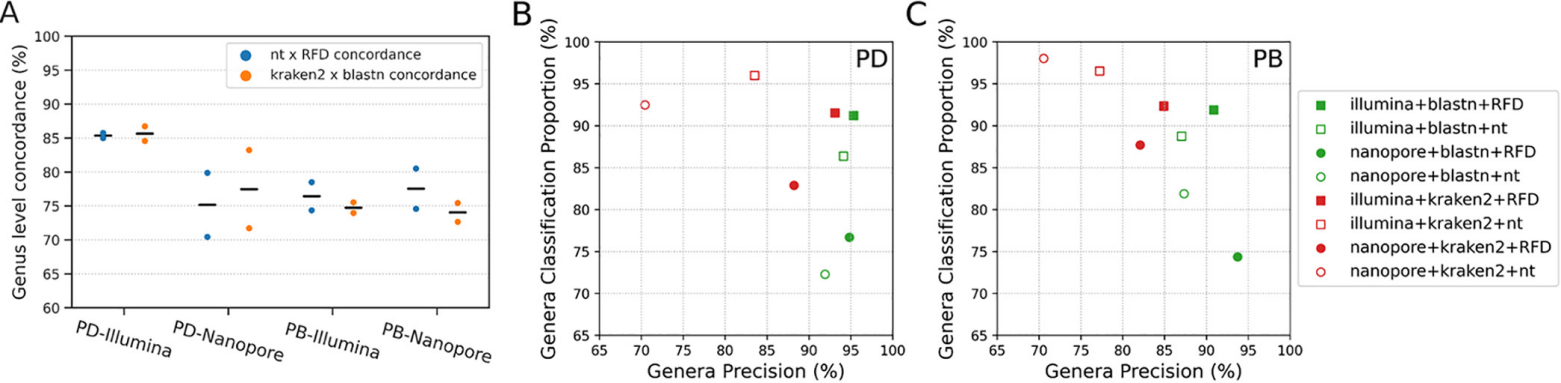
Analysis of shotgun metagenomics data. (A) Swarmplot showing the concordance in genus identification after varying either the alignment algorithm or querying different databases on different data inputs. nt = NCBI nucleotide database (29); RFD = RefSeq Fungi database (30); data inputs are indicated below the line (PD = pooled DNA; PB = pooled biomass); (B) Identification of fungal genera from PD samples. The classification proportion and precision were derived from different combinations of search algorithms and databases as indicated (box); (C) Identification of fungal genera from pooled biomass samples. The classification proportion and precision were derived from the different combinations of search algorithms and databases as indicated.

We compared different analysis strategies for each metagenomics shotgun data set. For nanopore data sets, we directly used the quality-controlled reads for classification. For Illumina data, we quality filtered all reads and assembled them into contigs before classification to maximize the classification accuracy. We performed both alignment and k-mer based classifications on these data using BLAST and Kraken2 (19, 21) using a “winner takes all” strategy in which the top hit was taken to assign the taxonomic classification of the query sequence. For each algorithm, we compared the use of two reference databases: the nonredundant NCBI nucleotide database (29) and the RefSeq fungi database (30) which only contains curated fungal genomes. We first assessed the performance of each alignment tool on both databases for each data input. We compared the concordance in the results of each pipeline at the genus level. We define concordance as the percentage of fungal genera identified by both analyses in a pairwise comparison (Fig. 1A). The concordance between analyses on each data set varied between 69% and 86% and generally, Illumina data resulted in a higher concordance than the nanopore data.

We then aimed to identify the combination of algorithm and database that yielded the most accurate species identification. We used classification proportion and precision to evaluate each classification, where $\text{Classification Proportion}=\frac{\#\;\text{total}\;\text{basepairs}\;\text{classified}.\;}{\#\;\mathrm{total}\;\text{basepairs}\;\text{of}\;\text{input}\;\text{reads}}$, and $\text{Precision}=\frac{\#\;\mathrm{total}\;\text{basepairs}\;\text{classified}\;\text{correctly}}{\#\;\text{total}\;\text{basepairs}\;\text{classified}}$.

The number of total base pairs is calculated as total read length for nanopore reads and total coverage of Illumina reads to each contig (24, 25). We plotted the precision and classification proportion for each pipeline and found three regular patterns (Fig. 1B and C): First, for each data set, BLAST resulted in higher precision but lower classification proportion by comparison to Kraken2. Second, Illumina contigs returned a higher classification proportion and precision than nanopore reads. Third, classification against the Refseq fungal database yielded higher precision than those against the NCBI nucleotide database. In summary, BLAST alignments against the Refseq fungal database yielded the best classification strategy for shotgun metagenomics data sets. Interestingly, this result contrasts to the method of choice for amplicon data sets, in which the use of a restricted database generates higher false positives (27, 31).

### Applying cutoffs to query coverage improves classification accuracy on shotgun metagenomics data sets.

**FIG 2:**
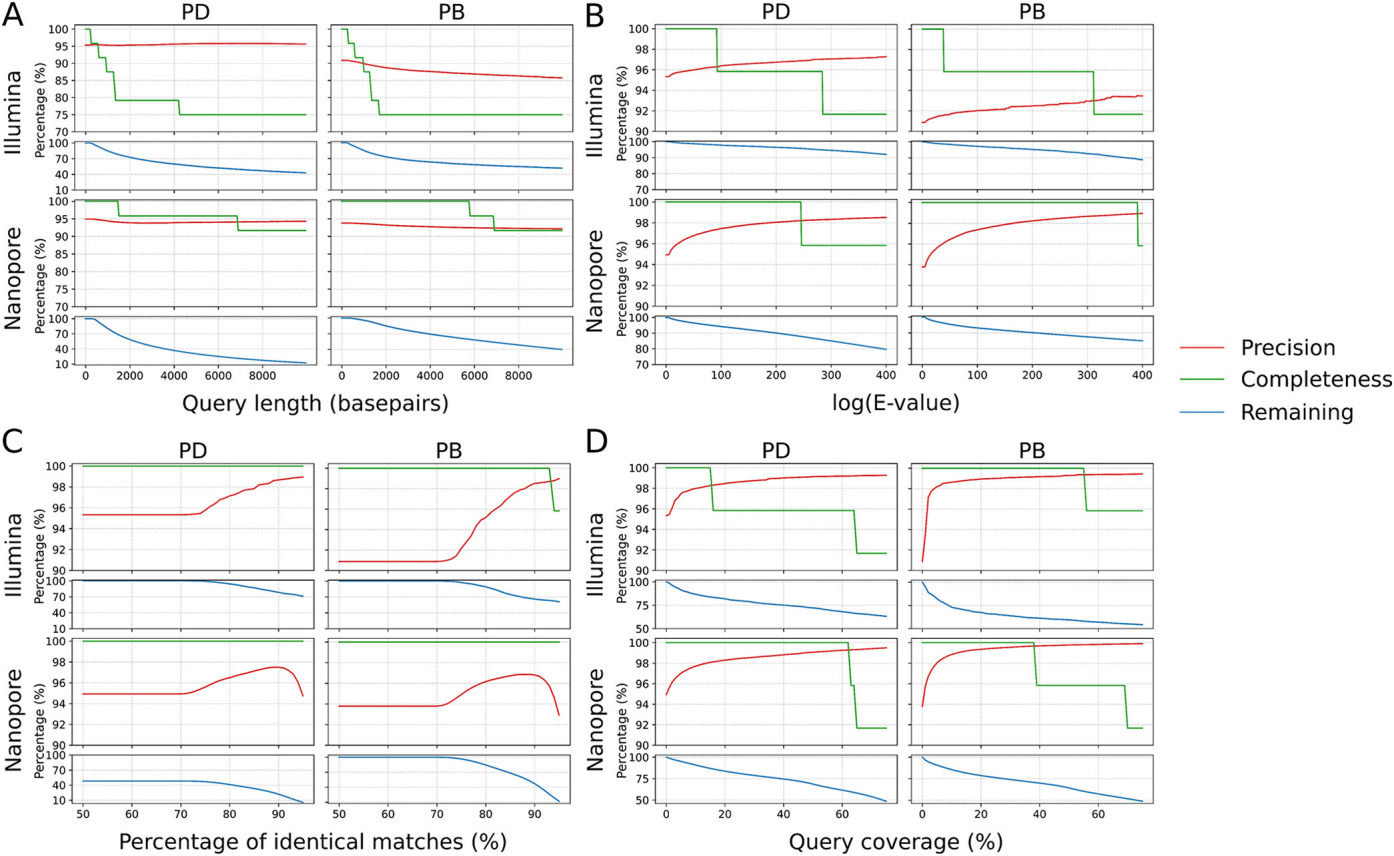
Dynamics in precision, completeness, and remaining rate after applying progressive cutoffs on BLAST alignment metrics. PD = pooled DNA; PB = pooled biomass. (A) Cutoffs applied to query length. (B) cutoffs applied to alignment E values. (C) cutoffs applied to the percentage of identical matches. (D) cutoffs applied to query coverage.

We next aimed to improve our classification scheme by filtering the BLAST search output. We reasoned that restricting alignment metrics would reduce the number of false classifications. To investigate changes in classification accuracy after restricting BLAST output parameters, we first BLASTed shotgun metagenomics reads against the RefSeq fungi database without applying any filter, then applied progressive cutoffs on different parameters of the BLAST results. We evaluated changes in the results based on the three metrics: precision, remaining rate, and completeness. Precision is described above and estimates the accuracy of the classification; remaining rate captures the percentage of the input data remaining after the application of each cutoff; and completeness is the number of taxa captured relative to the total number of taxa within the mock community. We initially applied cutoffs on query length; E value - the number of expected hits of similar quality that could be found by chance alone; and pident——the percentage of identical matches within the region of alignment between query and subject. As shown in Fig. 2A, applying progressive cutoffs to query length did not improve the precision, while both completeness and remaining rate diminished dramatically from very small cutoff values. Cutoffs applied to alignment E values removed <20% of the BLAST results, whereas precision showed minor improvement, especially on nanopore data sets (Fig. 2B). For Illumina data, applying cutoffs to the E value increased the precision by around 2% but at the cost of diminished completeness. E value cutoffs performed better on nanopore data sets, improving precision by 3% (pooled DNA) or 4% (pooled biomass) with non-identification of only a single genus from the mock community, at 10^−250^ or almost 10^−400,^ respectively. Progressive cutoffs on pident yielded the best results of all three filters. For Illumina data, precision was improved by up to 8% for data from pooled biomass sample, and completeness remained at 100% in almost all cases (Fig. 2C). For nanopore data sets, pident cutoffs improved the precision by up to ∼3% before sharp decreases with a concurrent filtering of ∼60% BLAST result as shown by the remaining rate. We think that these sharp decreases of precision are correlated with the nanopore error rate of 10% at the time. Given the characteristically high error rate of nanopore reads, we also applied cutoffs on quality scores to these data. Cutoffs applied to Phred scores did not alter the precision, while a significant proportion of the data set was lost through filtering (Fig. S1). Overall, our results suggest that applying E value and pident filters to BLAST results perform well on either Illumina or nanopore data but not both, and that cutoffs based on query length or quality scores did not affect the precision significantly.

10.1128/mBio.02444-21.2 FIG S1 Change of alignment metrics after applying cutoffs on Phred score. Download FIG S1, JPG file, 0.6 MB. Copyright © 2022 Hu et al. 2022 Hu et al. https://creativecommons.org/licenses/by/4.0/ This content is distributed under the terms of the Creative Commons Attribution 4.0 International license.

Given the results above, we investigated how the alignment parameters were calculated and explored other variables to improve the classifications. The BLAST E value is calculated as E = mn2^-S^ in which S is the bits score derived from the number of gaps and mismatches in the alignment, and m and n are the query length and database total length, respectively (32). Therefore, the E value is influenced exponentially by the alignment quality. We next investigated query coverage, a metric based on how much of the query sequence aligned to the subject. We calculated the query coverage as the number of identical matches divided by the read or contig length and applied progressive cutoffs on this parameter for each data set/algorithm analysis. As shown in Fig. 2D, applying cutoffs on query coverage improved the precision of all four analyses significantly, and did not cause losses of completeness at smaller cutoff values. For example, at a 10% cutoff on query coverage, the precision of all four analyses was 98–99% while the completeness remained at 100% and the remaining rate dropped by 10–25%. This result not only supported our hypothesis that the total length of the alignment matters as much as the alignment quality, but also suggested a novel approach to improve the accuracy of fungal classification.

### Improving taxa identification from published metagenomics data sets using query coverage as a filtering parameter.

After improving classifications by applying cutoffs to the query coverage on the mock community data sets, we extended this strategy to try to improve the classification of published shotgun metagenomics data sets. We reanalyzed 10 nanopore and six Illumina shotgun metagenomics data sets (33–36). These included host-associated fungal samples (nanopore) and host-depleted microbiome data (Illumina). For unified comparison, we used the BLAST algorithm for all samples, and the choice of database was primarily based on the specific aim of each study. For example, for all Illumina data sets, we downloaded the quality-controlled sequences and reanalyzed them using the assembly and BLAST pipeline described above against the NCBI nucleotide database since they investigated the total microbiome diversity of all species. For the nanopore human data sets (35), we used the BLAST results taken directly from the original articles for analysis focusing on the NCBI nucleotide database. For the infected wheat data sets (34), we downloaded the sequences and reanalyzed them against the RefSeq fungal database as the aim of this study is to identify a limited number of fungal pathogen.

Since the environmental data sets contain unknown species, we followed the concept of precision to evaluate the classification of each sample. We calculated the percentage of the data set that was classified into taxa known to be included in the sample. For example, in reanalyzing human clinical samples (35), we included the pathogen (Pneumocystis jirovecii) and the human host (Homo sapiens) as the known taxa and calculated the total proportion of query sequences classified to these taxa before and after applying cutoffs on query coverage. Table 3 shows the improvement in taxonomic classification from the published data sets after applying query coverage cutoffs. We initially applied a 20% cut-off on the query coverage for all analyses, but the data loss in most cases was too high. Therefore, we applied query cutoffs that filtered around 20% of the BLAST results (20% drop of remaining rate) based on our analysis of the mock fungal community data sets (Fig. 2D). The percentage of confirmed genera increased for nearly all data sets after applying query coverage cutoffs (Table 3). For the Illumina microbiome data sets, we first assessed the change of proportions in fungal taxa after applying cutoffs on query coverages using the species lists identified by Donovan et al. (37) as confirmed taxa. We observed only marginal increase in percentages for the confirmed fungal communities, due to their low total proportions in the original samples. We then calculated the improvement in precision for the bacterial communities. The Illumina data sets were generated from swine and mouse gut microbiome samples, so we assessed the change in proportions of their core bacterial genera (a group of bacteria commonly present in swine and mouse guts [38, 39]). The percentages of confirmed core bacterial genera improved by up to 5.7% after applying cutoffs on query coverage (Table 3). In addition, in the nanopore human data sets, the total percentage of reads classified as *Homo* sp. in the three healthy individual samples were improved by applying cutoffs to query coverage. These results indicated that this strategy may be broadly applicable not only to fungal species, but also to the classification of other eukaryotes and bacteria. One Illumina data set (d1) and one nanopore data set (a5) showed decreased percentages of confirmed taxa after applying query coverage cutoffs, which might be because the core microbiome species are not representing the species identified in the Illumina sample, or due to the low coverage and high error rate of nanopore data.

**TABLE 3:** Assignment of published sequence data to genera after application of cutoffs to query coverage

Sample ID	Sample description	Sequencing tech	Cutoffs on query coverage (%)	Filtered results (%)	Percentage of confirmed genera before applying cutoffs (%)	Percentage of confirmed genera after applying cutoffs (%)
a1	Human sputum samples^35^	Nanopore	59	20.2	85.9	86.5
a2			53.2	20.1	97.9	98.5
a3			54	20.5	96.5	97.4
a4			45.5	20.1	16.2	19.8
a5			58.5	20	71.1	66.9
a6			50.4	20.1	93.6	94.7
b1	Field infected wheat samples^34^		5	20	60.4	75.1
b2			0.77	19.9	34.8	43
b3			12	19.7	67	82
b4			0.61	20	5.8	6.2
c1	Swine gut microbiome samples^38^	Illumina	2.4	20.1	32	35.4
c2			3.3	20.2	34.2	36.6
c3			2.6	20.2	35.2	38.3
d1	Mouse gut microbiome samples^39^		3.4	19.8	29.1	24.3
d2			14	20.1	63.7	69.4
d3			4.5	20.2	38.6	42.3

### Benchmarking classification pipelines for amplicon data sets identified advantages of each strategy.

**FIG 3:**
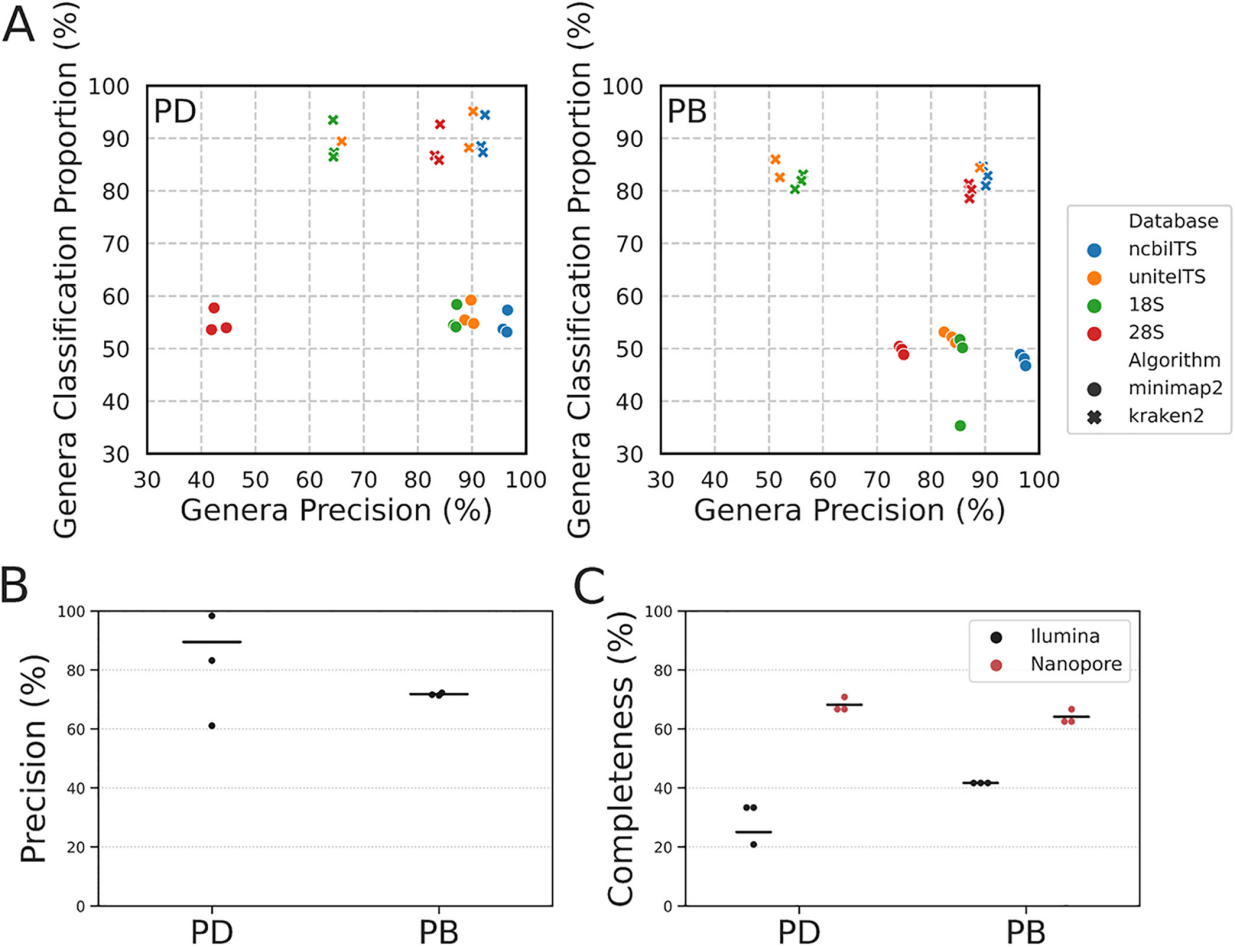
Benchmarking of amplicon data sets. PD = pooled DNA; PB = pooled biomass. (A) Scatterplot representing genus level classification proportion and precision for nanopore amplicon data. (B) Genus level precision of Illumina amplicon data. Classification proportion values for Illumina data were 100% due to the nature of the QIIME2 pipeline (based on the UNITE ITS database). (C) Genus level completeness of both nanopore and Illumina amplicon data sets. The nanopore results are from minimap2 algorithm against the UNITE ITS database.

We next assessed different strategies for the classification of ITS amplicon data sets. We amplified the ITS region from both mock communities using two different primer pairs and three technical replicates for each sample. Taking advantage of nanopore technology, we performed long-amplicon sequencing of a roughly 3 kb rRNA gene region covering part of the 28S subunit, ITS1, 5.8S subunit, ITS2 and part of the 18S subunit (11). For Illumina sequencing we used the well-established ITS1F-ITS2 amplicon of about 300 bp in length (40). Similar to the analysis of the shotgun data sets, we applied both k-mer and alignment-based approaches to the classification of nanopore amplicon data. We used the pairwise alignment algorithm minimap2 as the alignment algorithm instead of BLAST due to its speed and efficiency. We tested four different databases for classification of long amplicons; the NCBI 18S and 28S databases, and two ITS databases from NCBI and UNITE (30, 41). Overall, we found that the k-mer algorithm returned much higher classification proportion than alignment for each nanopore data set, but the highest precision (∼97%) values were achieved by combining the minimap2 alignment algorithm with the NCBI ITS database (Fig. 3A). For Illumina amplicon data sets, we applied the QIIME2 pipeline which is one of the most widely used strategies for ITS classification and community composition analysis (42). The QIIME2 pipeline groups similar Illumina amplicons into sequence features before classification to reduce the demand on computational resources (43). Since all individual Illumina reads are grouped into sequence features and all the sequence features are classified, the classification proportion of the Illumina amplicon data sets are 100%. We plotted precision rates from the QIIME2 analysis of both the pooled DNA and pooled biomass samples with their means (Fig. 3B). The mean precision values from either Illumina data set were lower than that from k-mer analysis of the respective nanopore data sets.

Although the precision values from amplicon data sets were higher than those from shotgun data sets, the ITS classifications did not identify all genera within the mock community, as shown by our completeness analysis (Fig. 3C). The nanopore amplicons identified 68% (pooled DNA) and 63% (pooled biomass) of the total genera in the mock community, whereas the Illumina amplicon data sets covered only 25% and 41% of the genera, respectively. We suspect that the low completeness from ITS classifications was due in part to the low quality of this particular data set (Table 2) and partially due to biases arising from non-uniform amplification by used primer pairs and different amplicon lengths. However, there were fewer nanopore amplicon reads than in the Illumina amplicon data sets and the completeness from the nanopore data were higher (Fig. 3C). This supports the argument that long amplicons identify a wider range of species and are more accurate in species classification than short amplicons (44, 45).

### Cutoffs on query coverage also improve community composition analysis.

**FIG 4:**
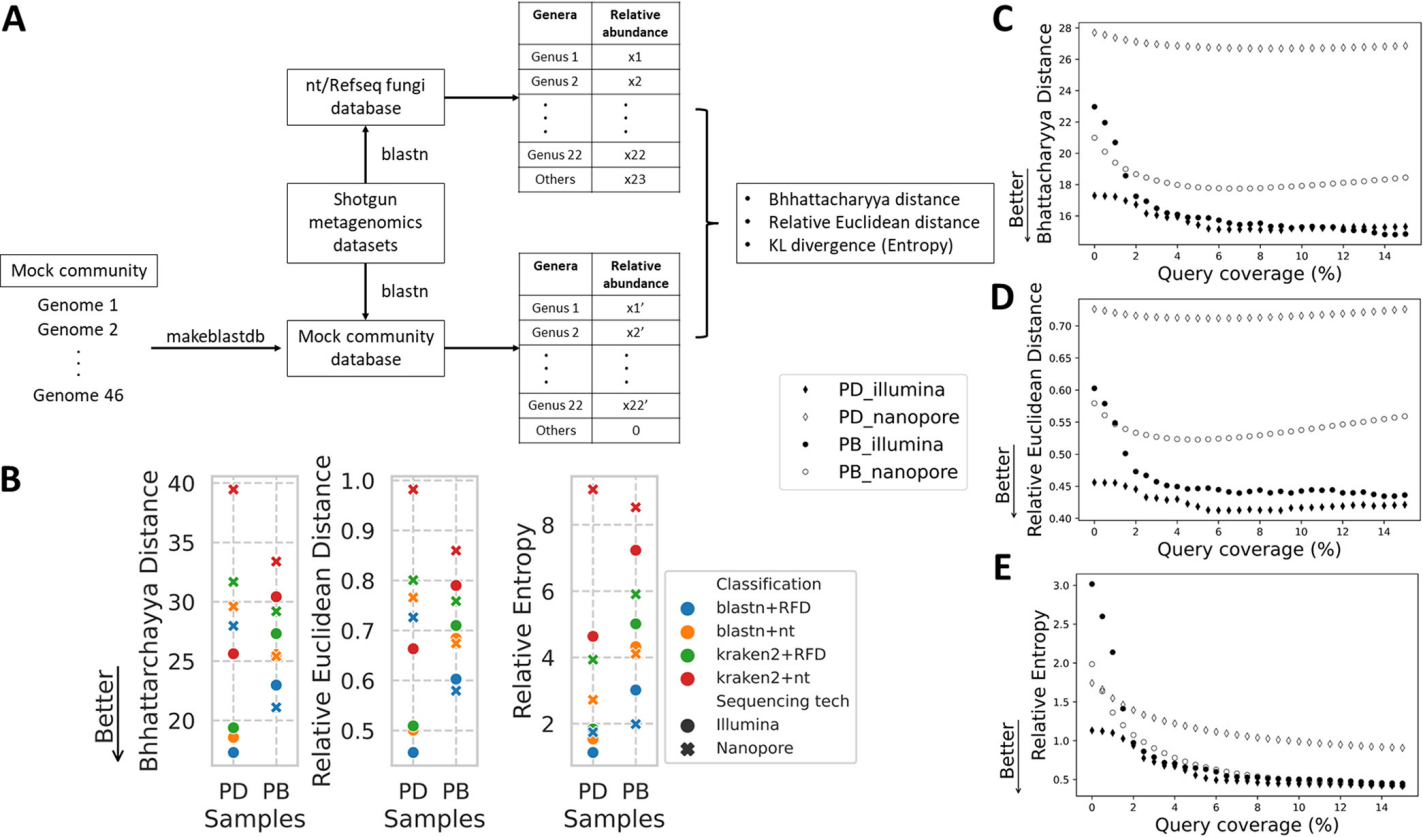
Improving community composition analysis by applying query coverage cutoffs. PD = pooled DNA; PB = pooled biomass; nt = NCBI nucleotide database (29); RFD = RefSeq Fungi database (30) (A) Experimental flowchart for analyzing community compositions. (B) Statistical similarity measures between gold standard community composition and each combination of algorithms and databases. Lower values correspond to greater similarity between the samples and the gold standard. (C) Change in Bhattacharyya distance after applying cutoffs to query coverage for each data set as indicated. The query coverage gap between each dot point is 0.5%. (D) Change in relative Euclidean distance after applying cutoffs to query coverage for each data set. The gap between each dot point is 0.5%. (E) Change in relative entropy after applying cutoffs on query coverage for each data set. The gap between each dot point is 0.5%.

We next calculated community compositions using the results from BLAST search against Refseq fungal database for all shotgun metagenomics data sets. Community composition refers to the identity and relative abundances of all taxa in a community. Given the observation that use of a restricted database resulted in higher classification precision, we constructed a database containing only the genomes from species within the mock community and aligned all data to the mock community database using BLAST. This forces the precision to 100% as any classification will belong to a species from the mock community. We then BLASTed each data set against this database and calculated the relative abundance of each genus. We defined this as the ‘gold standard’ for community composition analysis of the mock fungal community (Fig. 4A). We then compared the community composition determined from each combination of algorithms and databases with the gold standard for each data set, and measured their differences using three statistical distance tests: Bhattacharyya distance, relative Euclidean distance and relative entropy (46–48). Consistently, BLASTing sequences against the Refseq fungal database produced community compositions with the highest similarity to the gold standard analysis (Fig. 4B).

To assess whether query coverage cutoffs also improved the community composition analysis of shotgun metagenomics data, we plotted the changes in statistical distance after progressive application of query coverage cutoffs (Fig. 4). After applying cutoffs on the query coverage, the community composition improved in all cases especially for lower cut-off values. The community compositions from pooled DNA Illumina data sets improved and turned out to be the most similar result to the gold standard at query-coverage cutoffs greater than 3–4%, which is consistent with the changes in precision rate shown in Fig. 2D. Overall, our results illustrated that applying cutoffs on query coverage did not only improve the classification accuracy, but also the community composition analysis.

## DISCUSSION

Here, we investigated the taxonomic classification of DNA sequences, one of the key steps in all metagenomic workflows, with a particular focus on fungi. After assessing various combinations of algorithms and databases following different sequencing strategies, we found that combining BLAST with the fungal specific Refseq fungal database always resulted in the most precise classifications for all mock fungal community data sets. These classifications were further improved when applying cutoffs on query coverage, including positive flow on effects on downstream community composition analysis from shotgun metagenomics data sets.

Although sampling strategies and DNA extraction substantially influence the outcome of species classifications (49–51), choosing an appropriate sequencing strategy is a major step toward accurately profiling a sample. Overall, our results suggested that both short- and long-read shotgun metagenomics data sets have comparable accuracy, and both achieved higher accuracy than amplicon data sets. However, Illumina shotgun data sets require additional steps to assemble reads into contigs before querying them against a database, and to map all reads back to the assembly to quantify the coverage. These processes are necessary to achieve accurate classification from longer contigs (52), but take longer than analysis of long-read shotgun data. For amplicon data, long range amplicons performed better than short ITS data in classification accuracy and completeness (Fig. 3), consistent with other studies (44, 45). The overall completeness from analyzing amplicon data sets is much lower than from shotgun data sets (Fig. 3C). This may be because we used far less amplicon data for benchmarking classification pipelines, and an incomplete database which does not contain all taxa present in our mock community. On the other hand, amplification biases are another major source of poor community recovery. These biases come mainly from inconsistent amplification of the barcoding regions of different species, caused by copy number variations and different primer binding specificities (53, 54). Also, particularly for fungi, variations in barcode (amplicon) length are the major source of bias in recording fungal community compositions, with longer barcodes being underrepresented (55). Overall, analysis of the long-read shotgun data sets returned the most accurate fungal classification.

Next, we found that the alignment algorithm (BLAST) outperformed the k-mer based approach (kraken2) in classification accuracy (24, 56). We also compared the effects of progressive cutoffs to major alignment parameters for classification of shotgun metagenomics data. We found that applying read length or read quality cutoffs did not improve the precision of the classification for all shotgun data sets (Fig. 2A, Fig. S1). This observation contrasts with a previous study based on simulated data which claimed that the use of long reads improves the accuracy of classification (57). Cutoffs on pident slightly improved the classification accuracy for Illumina data sets, but the error-prone nature of the nanopore data (∼10% error rate with Guppy 4 basecalling) affected the results evident as a breakdown of precision when pident cutoffs reached 90% (Fig. 2C).

We found that query coverage cutoffs that filtered out ∼20% of BLAST results worked most effectively for improving the classification (Fig. 2D, Table 3). Unlike E value which weights gaps and mismatches as the major factors affecting alignment quality, query coverage weights the query length as well as the number of identical matches in assessing alignment quality. In this case, we can eliminate spurious alignments that are due to a small proportion of reads with high fidelity to the reference which are commonly present in metagenomics data sets. Interestingly, higher cutoffs on query coverage (10–20%) are observed from mock community data sets than real environmental data sets given the same 20% filtering threshold on BLAST results, including few extremely low thresholds of query coverage in the contigs from Illumina shotgun data sets. Other studies that used simulated data to generate metagenomics contigs for classification employed 90% query coverage cutoffs (58–60). This clearly indicates an important difference between real mock community data sets and simulated data based on reference databases, because contigs from simulated reads are artificially more similar to the reference genomes than the real data. Therefore, the use of simulated data may overestimate the specificity of the alignment, which leads to overoptimistic estimation of the classification. Together with the different results from read length and read quality cutoffs, these observations highlight the differences between the use of real environmental data and simulated data in benchmarking studies, especially for the classification of complex microbial communities.

There were some differences in results after analyzing pooled DNA and pooled biomass samples after comparing statistical distance with the gold standard. pooled DNA samples pooled DNA of individual species together while pooled biomass samples pooled equal amount of fungal tissue before the DNA extraction. After applying cutoffs on query coverage, both Bhattacharyya distance and Euclidean distance between the best practice (BLAST + Refseq fungal database) and the gold standard classification only showed marginal decrease in pooled DNA samples, and slowly reversed as the cutoffs increase (Fig. 4). In contrast, these distances from pooled biomass samples decreased more significantly and never reversed, indicating a clear improvement of the community composition analysis (Fig. 4). This may be because about 1/3 of reads were classified as *Candida* in the pooled DNA sample while only 10% reads were classified as *Candida* in pooled biomass sample. The difference in the relative abundance of this single genus between the community compositions of the gold standard and the best practice is much higher than that of other low abundance genera, and is thus more influential in calculation of the overall statistical distance of each data set.

Following the effectiveness of the query coverage, the next question is how to bring low quality but high coverage alignments into consideration? The winner-takes-all selection strategy could itself be redesigned, as highly conserved genome regions from closely related species often resulted in very similar alignment scores between the best alignment and other top alignments of each query. In this case, a weighting statistic and the relative probabilities of multiple top taxonomic assignments can be explored to replace the best-hit-takes-all strategy. This will be particularly useful in conjunction with the rapid expansion of fungal genome databases.

Next to the use of the best classification tool, choosing the most appropriate database significantly influences analysis outcomes (25, 26). Based on our observations, we suggest that ‘prior knowledge’ of the data set should guide the choice of database as this will improve the accuracy of taxonomic classifications. For example, our results suggested that the restricted database resulted in more accurate fungal classifications for shotgun metagenomics data sets. This strategy might be appropriate if queries are initially binned into kingdoms before a more in-depth analysis using kingdom specific databases. Also, Kaehler et al. (61) incorporated environment-specific taxonomic abundance information into the analysis of amplicon data sets and showed that these improve classification accuracy. Similar approaches can be applied to metagenomic data sets. In addition, machine learning strategies are becoming increasingly popular for analyzing genomic data. Here, taxonomic classifiers could be trained on classified sequence data sets before being applied to communities with similar compositions to the training data sets, or to identify target species from complex communities (56, 62).

## MATERIALS AND METHODS

### Code availability.

All detailed commands and scripts used in each step are summarized in https://github.com/Yiheng323/Inferring-species-compositions-of-complex-fungal-communities-from-long--and-short-read-sequence-data.

### Fungal harvesting, DNA extraction and construction of mock communities.

Selected fungal strains were grown for 48 h at 27°C on Sabouraud dextrose agar. For the species in the pooled DNA community, an inoculation loop of fungal cells was scraped into a 1.5 mL microcentrifuge tube and ground with a pestle in liquid nitrogen. Genomic DNA was extracted using the Zymo Research *Quick*-DNA Fungal/Bacterial Miniprep kit (cat. no. D6005 Zymo Research, Irvine, CA, USA). First, BashingBead Buffer was added to the ground fungal cells and vortexed. The mixture was filtered through a Zymo-SpinTM III-F Column and the filtrate was combined with Genomic Lysis Buffer. The mixture was filtered through a Zymo-Spin IICR Column and washed with DNA Pre-Wash buffer and g-DNA Wash Buffer. The DNA was eluted in nuclease-free water. DNA concentrations were measured using the DeNovix dsDNA Broad Range kit (DeNovix, Wilmington, DE, USA) and 250 ng of DNA from each strain was then pooled to create the final community.

For the pooled biomass community, two inoculation loops of fungal cells of each species in the mock community were scraped into a ceramic mortar. Liquid nitrogen was poured into the mortar and the fungal mixture was ground into a fine powder. DNA was extracted using the Qiagen DNeasy PowerMax Soil kit (cat. no. 12988-10 Qiagen, Hilden, Germany). PowerBead Solution and Solution C1 were added to the ground fungal material, vortexed and centrifuged. The supernatant was then added to Solution C2, mixed and centrifuged, which was then repeated with Solution C3. The resulting supernatant was combined with Solution C4 and centrifuged through a column. The column was then washed twice with Solution C5. Final DNAs were eluted in nuclease free water and the concentration measured using the DeNovix dsDNA Broad Range kit.

### Library preparation and sequencing.

The ITS1 regions of the rRNA gene were amplified with the universal fungal primers, ITS1F (CTTGGTCATTTAGAGGAAGTAA) and ITS2 (GCTGCGTTCTTCATCGATGC) (40). Sequencing of PCR amplicons was conducted on the MiSeq System (Illumina, San Diego, CA, USA) at the Australian Genome Research Facility. The Illumina bcl2fastq 2.18.0.12 pipeline was used to generate the sequence data. Paired-end reads 2 × 300 bp were generated up to 0.15 GB per sample for amplicon data. The Illumina amplicon data were then imported directly into QIIME2 for analysis. For shotgun Illumina data sets, we employed the same sequencing pipeline as the amplicon data, with MiSeq and the bcl2fastq 2.18.0.12 pipeline for the 2 × 300 bp paired-end reads. Raw shotgun Illumina reads were trimmed of adapters with Trimomatic (63). Quality controlled, paired-end reads were merged and assembled into metagenomics contigs using IDBA_UD (64), which is suitable for data sets with uneven sequencing depths of each species. After assembly, raw reads were mapped back to the contigs using bwa-mem (65), and the bam files were generated and sorted from sam files using samtools (66). Bedtools (67) was used for generating coverage for each contig, and we used python numpy and pandas module to calculate the average coverage for each contig.

For Nanopore sequencing (both shotgun and amplicon sequencing), we used Ligation Sequencing 1D SQK-LSK108 and Native Barcoding Expansion (PCR-free) EXP-NBD103 kits from ONT (UK), as adapted by Hu and Schwessinger (68), which was modified from the manufacturer's instructions with the omission of the DNA fragmentation and DNA repair steps. DNA was first cleaned up using 1× volume of Agencourt AMPure XP beads (cat. no. A63881, Beckman Coulter, Indianapolis, IN, USA) following the manufacturer’s instructions. We then eluted the DNA from the beads in 51 μL nuclease free water and quantified it using NanoDrop and Quibit Fluorometers (Thermo Fisher Scientific, Waltham, MA, USA). DNA was end-repaired (NEBNext Ultra II End-Repair/dA-tailing Module, cat. No. E7546), cleaned up with1x volume beads (AMPure XP beads), and eluted in 31 μL nuclease free water. The barcoding reaction was performed by adding 2 μL of each native barcode and 20 μL NEB Blunt/TA Master Mix (cat. No. M0367) to 18 μL DNA, mixing gently and incubating at room temperature for 10 min. A 1× volume (40 μL) Agencourt AMPure XP beads cleanup was then performed, and the DNA was eluted in 15 μL nuclease free water. Ligation was performed by adding 20 μL Barcode Adapter Mix (EXP-NBD103 Native Barcoding Expansion kit, ONT, UK), 20 μL NEBNext Quick Ligation Reaction Buffer, and Quick T4 DNA Ligase (cat. No. E6056) to the 50 μL pooled equimolar barcoded DNA, mixing gently and incubating at room temperature for 10 min. The adapter-ligated DNA was cleaned-up by adding 0.4× volume (40 μL) of Agencourt AMPure XP beads, incubating for 5 min at room temperature and resuspending the pellet twice in 140 μL ABB provided in the SQK-LSK108 kit. The purified-ligated DNA was resuspended by adding 15 μL ELB provided in the SQK-LSK108 kit and resuspending the beads. The beads were pelleted again, and the supernatant sequencing library was transferred to a new 0.5 mL DNA LoBind tube (Eppendorf, Germany). Nanopore sequencing was carried out on a MinION MK1b device using R9.4.1 Flowcells. Raw fast5 files were barcode demultiplexed by deepbiner (ONT), then basecalled by Guppy (v3.6.0, ONT, UK). Quality passed reads in fastq files were trimmed of adapters and barcodes using qcat (ONT, UK). For the long amplicon data, we filtered out reads less than 2000 bp. All sequencing data were submitted to the NCBI Sequence Read Archive (SRA) under the Bioproject PRJNA725368 including eight accessions: SRX10705648, SRX10705649, SRX10705650, SRX10705651, SRX10705695, SRX10705696, SRX10705697 and SRX10705698.

### Genome assembly.

While generating the reference genome database we found that there were no reference genomes for *Diutina rugosa* (former *Candida rugosa*), *Diutina mesorugosa* (former *Candida mesorugosa*), and *Filobasidium magnus* (former Cryptococcus *magnus*), so we performed nanopore sequencing on pure DNA from each species and assembled their draft genomes. These assemblies were of sufficient contiguity and quality (Table S1), so we added the new draft genomes into the reference database.

The nanopore data for *Diutina rugosa*, *Diutina mesorugosa,* and *Filobasidium magnus* were generated individually using the Ligation Sequencing 1D SQK-LSK108 kit alone, and from independent flowcells. Data from each flowcell was basecalled and quality filtered using the same pipeline as described above. We got roughly 40× genome coverage for *Diutina rugosa* and *Diutina mesorugosa*, and 20× coverage for *Filobasidium magnus.* Draft genomes were assembled with Flye (69) using default parameters and an estimated genome size of 20 Mb. After assembly, the contigs were polished 10 times with Racon (70) using nanopore reads, followed by a single polishing step with Medaka (ONT). The polished assemblies were assessed for completeness using BUSCO (71). The assembly statistics were reported from Flye.

### Database constructions.

For shotgun metagenomics analysis, we used three BLAST database and three kraken databases. Two databases (NCBI nucleotide database and Refseq fungal database) are from the same NCBI source, downloaded in May 2019. BLAST and kraken2 nt databases were downloaded using the updateblastdb.pl script from BLAST+ package (72) and the kraken2-build command (21), respectively. The fasta files of the RefSeq fungal database were downloaded from NCBI and converted to a BLAST database using the makblastdb command from the BLAST+ package (72), and were added to the kraken2 database library using the kraken2-build command.

To generate the mock community database containing only those species in the mock community for BLAST, we downloaded the genomes of all mock community species from NCBI according to their accessions (Table 1) and concatenated them with the three newly assembled genomes of *Diutina rugosa*, *Diutina mesorugosa,* and *Filobasidium magnus.* Following the previous pipeline (73), we then performed a kraken2 search to identify the potential contaminated regions in the concatenated fasta and masked those regions using bedtools (67). This kraken2 search used the standard kraken2 database which was build using the kraken2-build command. We also masked the low complexity regions using dustmasker from the BLAST+ package (72). To enable new genomes to be indexed by blastn, we updated the taxonomic map file by adding the fasta headers of the three new genomes and manually assigned their taxonomic IDs in the file. Lastly, we used the makeblastdb program to construct the mock community database.

For amplicon data analysis, we used two versions of the fungal ITS database from NCBI and UNITE, plus the fungal 18S, 28S database from NCBI. All of these were downloaded in fasta format in February 2020 and added to the kraken2 database library using the kraken2-build command.

### Data analysis.

For shotgun metagenomics data sets, we first used blastn (version 2.10.1) and kraken2 (version 2.0.8) to assign the NCBI taxonomic ID for each Illumina contig or Nanopore read. During classification, we found that a potential contaminant species Purpureocillium lilacinum was present in all samples in significant abundance (10–20%). Therefore, we added this species to the species list. The best hit from BLAST, or the species with the highest k-mer counts for each read and/or contig, was retained for further analysis. After classification, we used the python pandas module to merge information from different output files, and used the ete3 module (74) to assign taxonomic information to each read or contig. The relative abundances of each classification were calculated based on the total length of nanopore reads or total coverage of Illumina contigs. We used the python numpy and math modules for all statistical analysis.

For amplicon data sets, we sequenced each sample with three technical replicates. The classification workflow was different for data sets with different sequencing technologies. We used only the QIME2 workflow plus the UNITE database for the Illumina amplicon data, since it is the widely used method for classification. The paired-end reads were denoised using the DADA2 (75) plugin and assigned taxonomic information using the q2-feature-classifier (76) plugin. The QIME2 classifier was trained by the database sequence before classification. The classification output .qzv files were visualized by the QIME2 view website (https://view.qiime2.org/) and the feature-frequency csv file was extracted from the website. We then used the python numpy and math modules for the mathematical analysis and used the seaborn module to generate figures.

For nanopore amplicon data sets, we used kraken2 as the k-mer based algorithm and minimap2 as the alignment-based algorithm. The kraken2 command is the same as the kraken2 analysis for the shotgun metagenomics data sets but uses different databases. For the minimap2 analysis, we extracted the accessions of the best hits from the output files, and searched their corresponding taxonomic IDs from the NCBI taxonomic map (downloaded from https://ftp.ncbi.nih.gov/pub/taxonomy/accession2taxid/nucl_wgs.accession2taxid.gz, in June 2020) using the python pandas module. We then merged information from different output files and used the ete3 module to assign taxonomic information to each read.

### Availability of data and material.

All sequencing data were submitted to NCBI Short Read Archive (SRA) under the BioProject PRJNA725368 including eight accessions: SRX10705648, SRX10705649, SRX10705650, SRX10705651, SRX10705695, SRX10705696, SRX10705697 and SRX10705698.

10.1128/mBio.02444-21.1 TABLE S1 Assembly statistics of the draft genomes of *Diutina rugosa*, *Diutina mesorugosa* and *Filobasidium magnus* in the mock fungal community. Download Table S1, PPTX file, 0.04 MB. Copyright © 2022 Hu et al. 2022 Hu et al. https://creativecommons.org/licenses/by/4.0/ This content is distributed under the terms of the Creative Commons Attribution 4.0 International license.
