# Supplementary figures and images for: Inferring Species Compositions of Complex Fungal Communities from Long- and Short-Read Sequence Data
Source: mBio. 2022 Apr 11;13(2):e02444-21. doi: 10.1128/mbio.02444-21 (PMC9040722; doi:10.1128/mbio.02444-21)

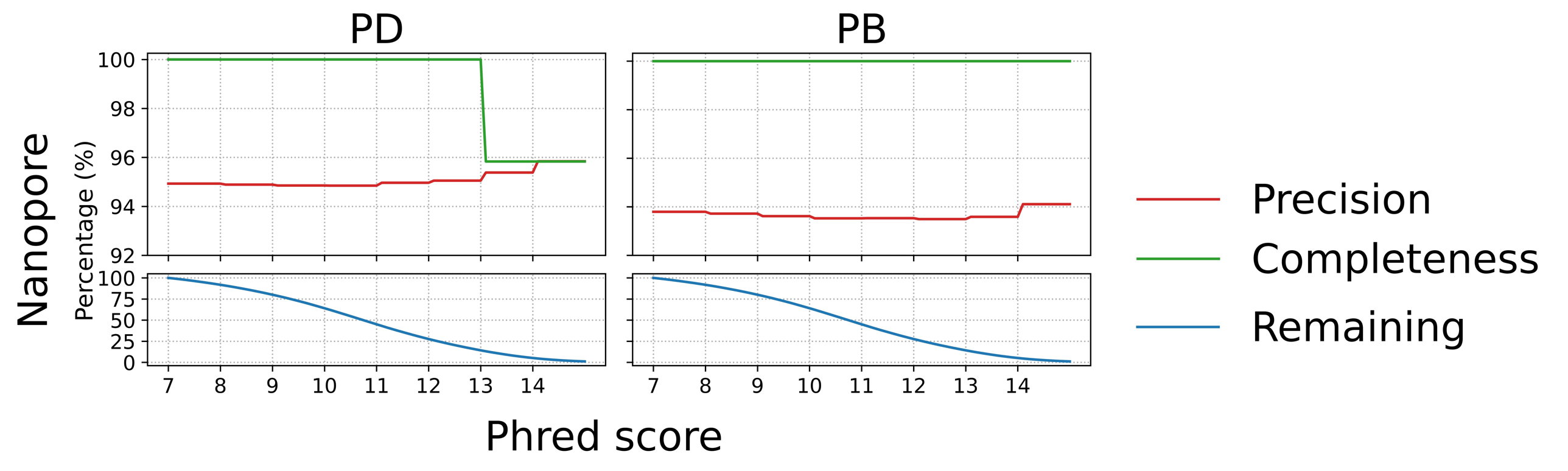

Supplement: FIG S1 [file mbio.02444-21-s0001.jpg]
