# Supplementary material for: Inferring Species Compositions of Complex Fungal Communities from Long- and Short-Read Sequence Data
Source: mBio. 2022 Apr 11;13(2):e02444-21. doi: 10.1128/mbio.02444-21 (PMC9040722; doi:10.1128/mbio.02444-21)
Supplement: TABLE S1 [file mbio.02444-21-s0002.pptx]

## Slide 1
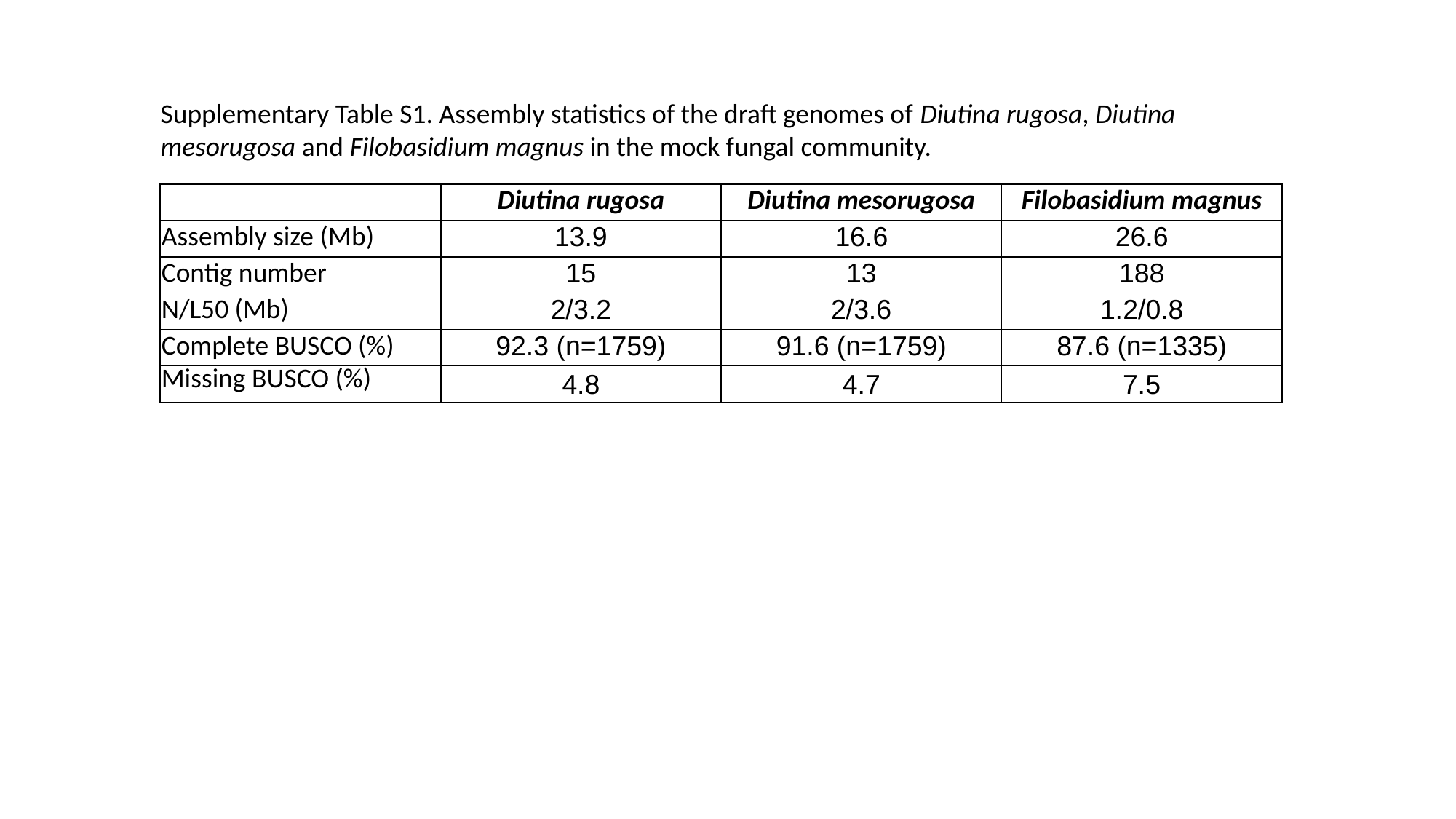

Supplementary Table S1. Assembly statistics of the draft genomes of Diutina rugosa, Diutina mesorugosa and Filobasidium magnus in the mock fungal community.
| | Diutina rugosa | Diutina mesorugosa | Filobasidium magnus |
| --- | --- | --- | --- |
| Assembly size (Mb) | 13.9 | 16.6 | 26.6 |
| Contig number | 15 | 13 | 188 |
| N/L50 (Mb) | 2/3.2 | 2/3.6 | 1.2/0.8 |
| Complete BUSCO (%) | 92.3 (n=1759) | 91.6 (n=1759) | 87.6 (n=1335) |
| Missing BUSCO (%) | 4.8 | 4.7 | 7.5 |
